# Clinical characteristics of asthma in the elderly in Argentina

**DOI:** 10.1186/1939-4551-8-S1-A108

**Published:** 2015-04-08

**Authors:** Anahi Yanez, Susana De Barayazarra, Marcela Soria, Edgardo Jares, Carlos Bueno, Nancy Recuero

**Affiliations:** 1 Inaer - Investigaciones En Alergia y Enfermedades Respiratorias, Argentina; 2 Servicio De Alergia e Inmunología Hospital San Roque, Argentica; 3 Hospital Zona General De Agudos Dr. Ricardo Gutierrez, Argentina; 4 Private Medical Centers SA, Argentina; 5 Universidad De Buenos Aires, Argentina; 6 Servicio De Alergia e Inmunología Hospital San Roque, Argentina

## Background

Few studies have focused on the characteristics of asthma in the elderly (AIE). Our study reflects the characteristics of old adults living in Buenos Aires who have been diagnosed of asthma.

## Methods

An observational descriptive study was performed at five different health care facilities in Buenos Aires. Clinical records during three months of 2014 were searched. Allergists reviewed all clinical histories and elderly was defined as older than 60 years. Clinical and laboratory characteristics were assessed, including severity of asthma (according to GINA), current treatment, evidence of sensitization via skin prick test (SPT), chest radiograph, total serum IgE and relative eosinophil counts.

## Results

Total 152 patients were included and their average age (SD) were 66.83 years (6.52), 73% women, 78% Caucasian and 22% Hispanic. Late-onset asthma (with asthma onset after 60 years old) was described in 10% of the patients, with mean (SD) age at diagnosis of 69,67 (6,81) years. Most of the patients had a diagnosis of persistent asthma (74%), while only a 7 % of them had a severe persistent asthma. They all were treated with an inhaled corticosteroid (ICS) + long-acting β-adrenergic (fluticasone + salmeterol (59%), and the first choice as acute reliever therapy was an inhaled fast-acting β-adrenergic agonist (salbutamol (89%). The doses of ICS were below 1000 mg/day in 77 % of the patients. Common sensitizing allergens (SPT) included mites (18%), pollens (8%) and fungi (7%). Anaphylactic triggers were predominantly tobacco smoke (40%), even though most of the patients had never smoked before (88%). Exercise (21%) and aeroallergens (20%) also triggered anaphylaxis. The chest radiograph was normal in the majority of the patients (89%). Elevated IgE and eosinophilia were only found in 4% and 30% of the patients, respectively.

## Conclusions

This is a retrospective study of AIE patients in Argentina. Better understanding of disease characteristics is needed to improve disease management.

